# Self-Supervision-Enabled Compounded Multi-Modal Feature-Learning Network for Classifying Depressive States with Fine-Grained Emotions Using Wearable Sensors

**DOI:** 10.3390/bios16050233

**Published:** 2026-04-23

**Authors:** Bhavani Ravi, Ibrahim Aljubayri, Usharani Thirunavukkarasu, Mohammad Zubair Khan

**Affiliations:** 1 Department of Computer Science and Engineering, Rajalakshmi Institute of Technology, Chennai 600010, India; srbhavaneesree@gmail.com; 2 Department of Computer Science and Information, Imam Mohammad Ibn Saud Islamic University (IMSIU), Riyadh 11564, Saudi Arabia; ialjubayri@outlook.com; 3 Department of Biomedical Engineering, Saveetha Engineering College, Chennai 602105, India; usharanithirunav@gmail.com; 4 Faculty of Computer and Information Systems, Islamic University of Madinah, Medina 42351, Saudi Arabia

**Keywords:** depressive state, emotional classes, deep learning, Galvanic Skin Response (GSR), ACC data, heart rate (HR)

## Abstract

Depression is a prevalent mental health disorder characterized by persistent sadness, loss of interest, and impaired daily functioning. Wearable monitoring systems have emerged as promising tools for continuous mental health assessment; however, they face challenges such as data privacy concerns, misclassification risks, and limited ability to capture complex emotional states. To address these limitations, this study proposes a Self-Supervision-Enabled Compounded Multi-Modal Feature-Learning Network (S2-CFL) for depressive state classification using wearable sensor data and psychological self-reports. The framework integrates a Twin-Path Encoder–Decoder Network (TP-EDN) for extracting temporal features from raw signals and a Densely Connected Convolution Pyramidal Transformer Network (DC2-PTN) for learning spatial representations from signal-to-image transformations. A fusion mechanism combines multi-modal features to predict depressive states, valence, and arousal levels, while a Fine-Grained Emotion Classification Network (FGECN) is employed to categorize emotional states into multiple classes using supervised learning models. Experimental results demonstrate that the proposed multi-modal approach improves classification performance and provides interpretable insights into emotional and depressive patterns.

## 1. Introduction

One of the world’s most prevalent mental health disorders is depression, which directly affects 300 million people across the world. Due to the recent pandemic issue, depression has increased by 30.7% on average. Due to fleeting emotional reactions [1], and fluctuations in mood, depression can differ among individuals for anhedonia, recurrence, and intensity. To be clear, depression is manifested by multifaceted interactions among biological, physiological, and socioeconomic factors, which can interact in an adverse feedback loop and be intensified by comorbid physical conditions [2]. However, those issues can be treated by robust methods such as antidepressant medication [3] and cognitive behavioral therapy. In addition to that, biomarkers can provide important data for diagnosing depression by analyzing heart rate (HR) variability and brain connectivity [4]. However, some social factors that hinder early interventions for affected individuals include high misdiagnosis rates, lack of health care providers, and inflated service costs.

There is need to develop a universal screening [5] approach for enhancing the fidelity of diagnoses. Continuous observation and the collection of psychological [6] and sensory data on daily patient activity also improves data utiliztion and cross-modal learning strategies. Utilizing advancements in machine learning (ML) and deep learning, recent studies have increasingly focused on self-supervised and multi-modal learning to improve depression detection accuracy [7], while multi-modal graph-based learning methods enhance cross-modal emotional understanding [8]. Furthermore, transformer-based architectures have shown superior capability in modeling multi-modal emotional states, and EEG-based deep learning models also capture subtle emotional variations effectively [9,10]. Some recent studies have also adopted advanced ML and DL techniques, along with passive modalities such as gyroscope readings, heart rate, posts on social media, smartphone text/call logs [11], and states of mental health, depression and health distress.

Screening for depression using passive smart phone data without affecting user privacy is desirable [12,13,14]. Data are acquired from varied wearable devices that also ensure objective and continuous monitoring of patients. However, combining wearable signs with physiological signals along with ML/DL techniques can predict anxiety and depression symptoms [15]. Personalized screening techniques have been introduced which could be provided to 2 billion people in 2025 with the extraction of advanced information such as message history [16] and location.

A multi-modal study combining sensors [17] and HR has provided valuable insights into the varied properties in depressive states. The transient changes in the sweat on the skin can be examined by skin conductance sensors [18], indicating mood changes and sympathetic activation of the ANS. The response of the autonomic nervous system (ANS), along with fight-or-flight response, is encapsulated in the HR information from wearable sensors. During the onset of depressive state symptoms, the quality of posture, stability, and balance are degraded, which leads to impaired psychomotor skills and a lapse into inactivity. To be more specific, the multi-modal technique monitors slight decreases in physical activity [19], which if left unmanaged can lead to moderate to severe depressive disorders.

**Figure 1:**
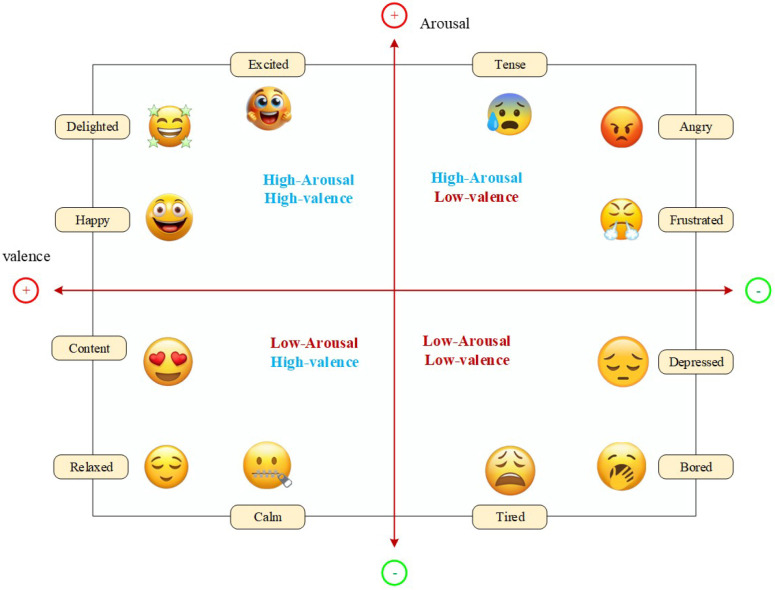
Valence − Arousal Effect Model of Russel.

Individual mental states cannot be completely inferred only by examining depressive states. Arousal, valance, and psychological effects are also essential [20]. The effect of negative and positive experience is assessed subjectively, measured by valance, the level of engagement and the activation of the sympathetic nervous system, which can be measured by arousal rate. Figure 1 shows the valance–arousal effects model.

With the help of body movements, psychological effects can be effectively monitored. Some gestures, such as shoulder shrugging, correlate with arousal/valance and motion, and minute movements can be continually monitored.

This study pioneered an unobtrusive monitoring technique that utilized body motion sole markers and wearable sensors to derive a comprehensive overview of mental stress states. The reason for adopting wearable sensors is that they can be used in naturalistic settings and accurately represent both behaviors and sub-conscious reactions. The main contributions of this research are listed as follows:

- **Self-Supervision-based Compounded Multi-Modal Learning:** The designed S2-CFL introduced a self-supervised learning method that firmly amalgamates wearable sensor data with psychological reports. The depressive state representations are autonomously learned from the multi-modal data sources without the need for manual annotations. Furthermore, the use of a Twin-Path Encoder–Decoder Network (TP-EDN) and Densely Connected Convolution Pyramidal Transformer Network (DC2-PTN) enables complementary feature extraction with better generalization capability, thereby detecting complex mental health states.
- **Multi-Level Fusion with Dense Connections:** With concatenation and dense connections, the features from the double paths (TP-DEN and DC2-PTN) are robustly correlated and fused. The multi-level fusion ensures that the different modality features (i.e., text-based reports and image-like representations of sensory data) are robustly combined with fine-grained and global emotional signals. This fusion network categorizes the users mental condition into three emotional dimensions, such as depression, arousal and valance.
- **Explainability-enabled Fine-Grained Emotion Classification Network:** The adoption of a Fine-Grained Emotion Classification Network (FGECN) provides categorization of depressive states into emotional classes such as calm, anger, sad, happy, excited, etc. …The FGECN is ensured by explainable ML algorithms that offer reliable and transparent decision making by resolving the common black box issues in conventional ML algorithms. The utilization of explainability guarantees the reliability and trustworthiness of the model’s predictions, which are then likely to be suitable for mental health diagnosis.

The proposed research article is organized as follows. Section 2 provides the materials and methods. Section 3 explores the results. Section 4 provides the discussion and findings. Section 5 concludes the research work with noteworthy future directions.

### Problem Formulation and Task Definition

The proposed framework is designed as a hierarchical multi-stage classification system that integrates multiple related tasks in a structured manner. In the first stage, the model performs three parallel binary classification tasks based on physiological and multimodal features:

- Depressive state classification (low vs. high).
- Valence classification (low vs. high).
- Arousal classification (low vs. high).

These three dimensions represent core affective states and are predicted using the S2-CFL architecture. In the second stage, the predicted representations from these affective dimensions, along with multimodal features, are used for fine-grained emotion classification. This stage categorizes emotional states into multiple classes, such as happy, sad, angry, calm, tired, and related categories.

## 2. Materials and Methods

**Figure 2:**
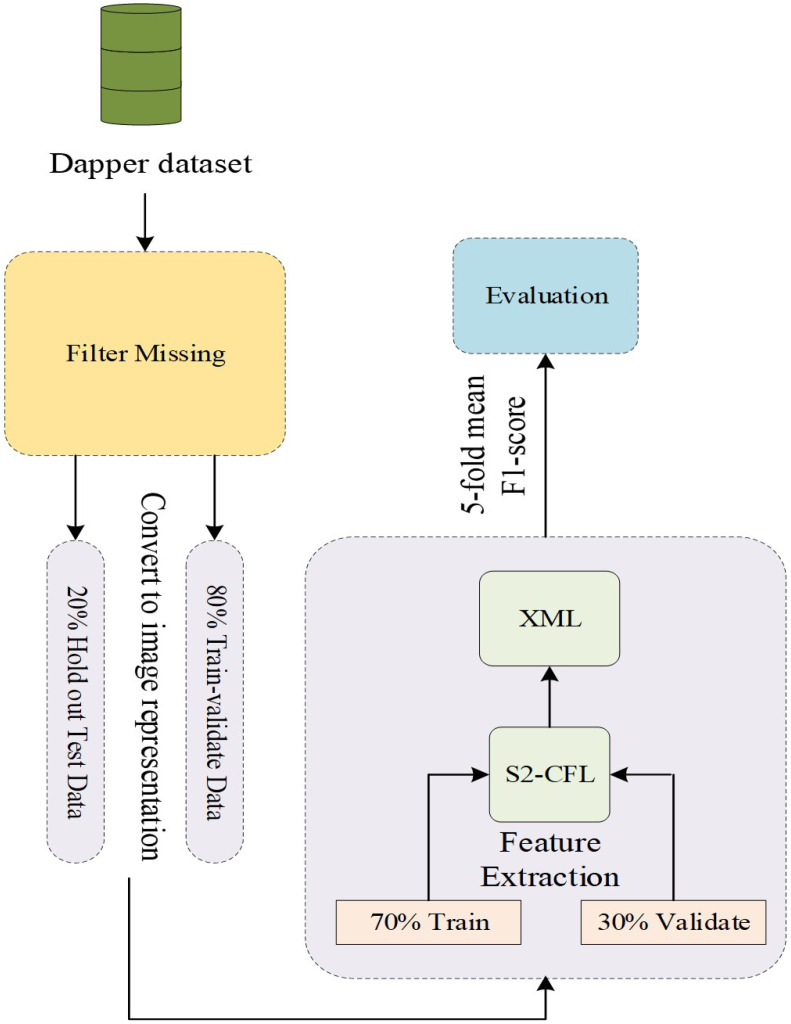
Pipeline of the proposed model.

The Daily Reconstruction Method (DRM) and Experience Sampling Method (ESM) ensure correlation among emotional states and mood (i.e., creativity). Nevertheless, those techniques are found lacking with regards to varied emotional states, due to physiological data scarcity. Figure 2 shows the overall pipeline of the proposed model, featuring data collection, processing, analysis, and assessment.

### 2.1. Dataset Details

The Daily Ambulatory Psychological and Physiological recording for Emotion Research (DAPPER) dataset, developed by researchers at the Institute of Psychology, Chinese Academy of Sciences (CAS), Beijing, China was utilized by our proposed research and provided vast amounts of physiological and self-reported psychological recordings from wearable bands and smart phones for daily emotional state exploration. The use of the adopted dataset also allows for naturalistic scenarios and omits lab-based predetermined experiments. Lab-based experiments although ensure correlation among physiological and psychological data but do not effectively simulate real-time day-to-day activities [21]. Existing lab-based experiments only utilize text, videos, and images for monitoring individuals over a limited period of time in laboratory settings. For the proposed research, data were collected by recording the day-to-day activities of participants over five consecutive days from 9.00 a.m. to 11.00 p.m. Table 1 shows details of DAPPER dataset contestants.

Although the DAPPER dataset provides realistic and naturalistic recordings, it is limited in duration to five consecutive days and has a sample size of 142 participants, which may restrict the generalizability of the proposed model to diverse populations and long-term behavioral patterns. For evaluation, the dataset was divided into training, validation, and testing sets using an 80:20 split strategy, which was followed by stratified 5-fold cross-validation to ensure robustness. To prevent data leakage and ensure reliable evaluation, a subject-independent splitting strategy was adopted, where data from the same participant does not appear across multiple splits, which avoids overestimation of performance due to subject-specific patterns. In terms of class distribution, the dataset exhibits moderate imbalance across depressive states and emotional categories, particularly between low/high arousal and valence classes, The evaluation protocol ensures that the reported performance reflects the generalization capability of the model rather than subject-specific characteristics. However, the dataset captures real-world physiological and psychological variations, making it suitable for initial validation.

**Figure 3:**
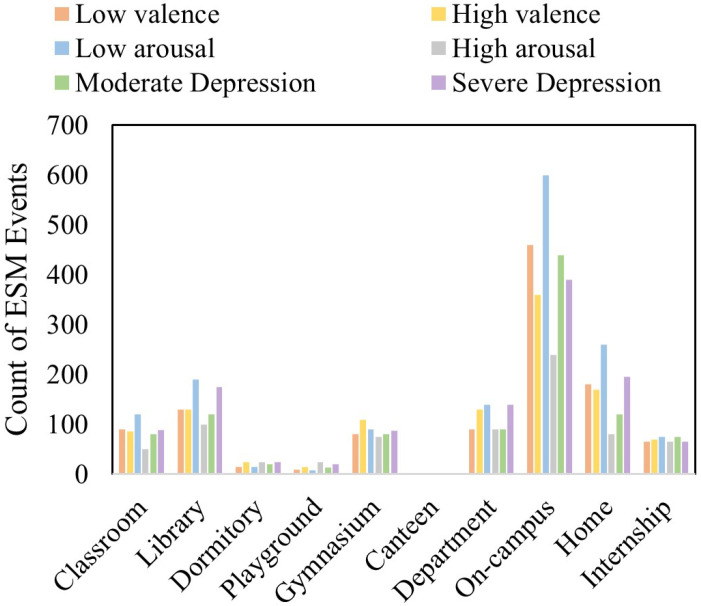
Environment setting based ESM count.

**Figure 4:**
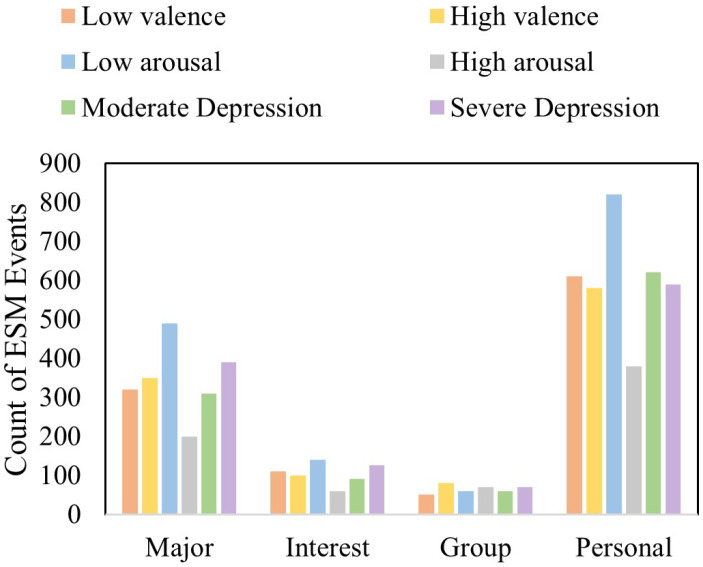
Activity-type-based ESM count.

Figure 3 shows the distribution of ESM events in settings such as department buildings, classrooms, and dormitories. The event frequency is also recorded and illustrated in Figure 4 which shows the relation of severe and moderate depression with low arousal, and also low and high valance.

The subjects were asked to perform the data collection procedure in the form of a pretest for examining their respective traits immediately before and after the experiments. With the help of the WJX survey platform, the pretest were conducted with sub-sections such as PANAS, BDI-II, etc. …

With the help of a Psychorus-manufactured wrist band, the physiological data were recorded. Utilizing a wrist-embedded accelerometer sensor, the ACC data were collected on *x*, *y*, and *z* axes at 20 Hz. In a similar manner, PPG data were also collected at 20 Hz with the green light of 532 nm. Furthermore, the GSR data were collected and sampled at a 40 Hz higher frequency. In order to minimize unwanted artifacts and noise and enable faster data representation and computation, the acquired signals were down-sampled on a 10 s time window at 1 Hz rms. The mean square trivial raw ACC and the PPG signals were down-sampled [22].

With the help of the Psychorus application, psychological recordings were obtained on the patients’ smart phones. The questionnaires were sent to the participants by considering DRM and ESM. The DRM questionnaires included the PANAS scale, arousal, and valance for describing the events. The questionnaire was sent to the patients every night at 11 p.m. The ESM questionnaire is sub-divided into the 10 item PANAS scale, arousal, and valence, the 10 item personality inventory (TIPI-C), and daily event information. Those data were provided to the patient at randomized time periods between 9.00 a.m. and 11.00 p.m. with a 90 min minimum break. The participants were advised to complete the questionnaire in 30 min based on the activities and events. For both the psychological recording and physiological recordings, 10 categories of emotions were chosen which included upset, hostile, alert, ashamed, inspired, nervous, determined, attentive, afraid, and active [23].

For a period of 5 days from Monday to Friday in the winter season, both psychological and physiological data were recorded. The patients included 78 females and 64 males with an age range of 18 to 31. A total of 142 patient records were obtained, while 54 patients were recorded using psychological data and 88 patients were recorded by physiological data. The local Ethics Committee at the Tsinghua university Psychology Department approved and ethical license based on the Helsinki Declaration standards [24].

**Figure 5:**
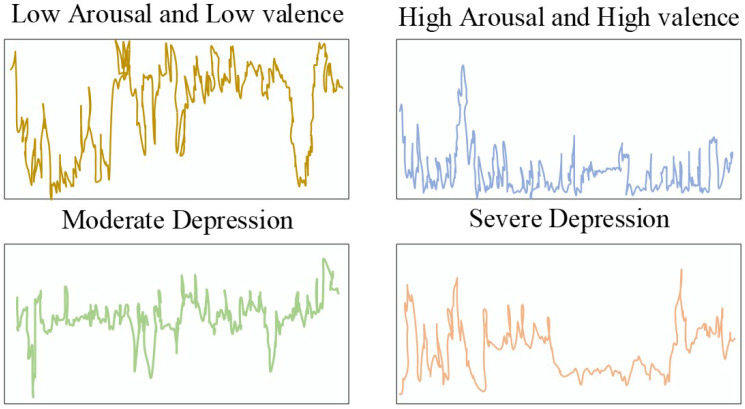
HR Instance on Binary Classes.

**Figure 6:**
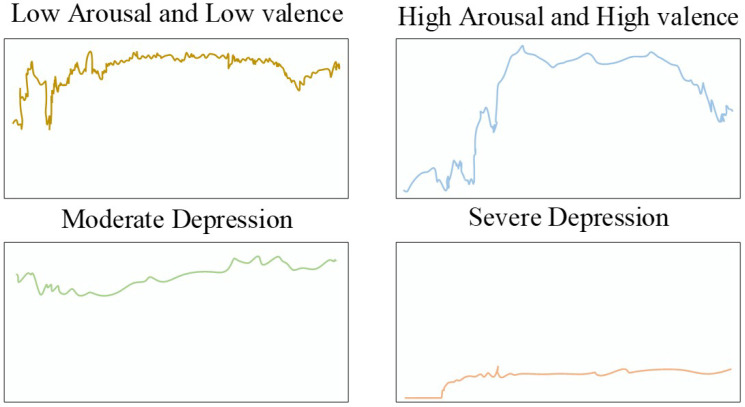
GSR Instance on Binary Classes.

**Figure 7:**
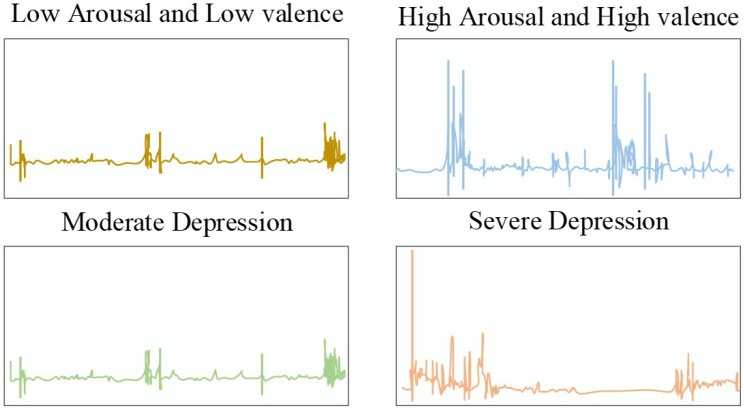
ACC instances for binary classes.

About 2249 signals with durations of 1713.4 s were recorded in the initial level of dataset formation. However, some of the signals were omitted because the ESM sampling method considers 30 min preceding the questionnaire. The omitted signals included ones with lesser duration and missing segments. Therefore, only 2035 signals were considering with durations of 1800.00 s with 30 min. Furthermore, the Z-score normalization method was utilized to resolve the amplitude scaling issue that is very common in wearable devices. Figure 5, Figure 6 and Figure 7 show the instances of samples from ACC, GSR, and PPG data.

### 2.2. Self-Supervised Learning Strategy

In this section, we explain the experimental results for depressive state severity quantification for every depression category, and emotion classification with unimodal and multi-modal categories. With the performance metrics accuracy, specificity, sensitivity, and F1-score, the proposed performance was analyzed. The proposed framework adopts a two-stage training strategy to ensure clarity and reproducibility. In the first stage, the TP-EDN module is pretrained using a self-supervised reconstruction objective, where the model learns latent representations by minimizing the Mean Squared Error (MSE) between the input physiological signals and their reconstructed outputs. In the second stage, the pretrained encoder weights are transferred to downstream classification tasks (depression, valence, and arousal) and fine-tuned using labeled data with the same optimization settings (Adam optimizer, learning rate 0.001). FGECN operates as a supervised module, utilizing CatBoost, XGBoost, and Random Forest on the learned feature representations, thereby clearly separating self-supervised feature learning from supervised classification.

### 2.3. Pre-Processing

**Figure 8:**
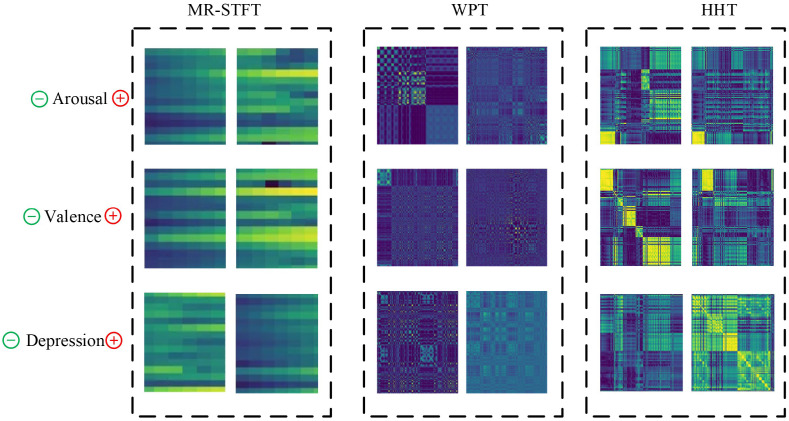
Signal to Image representation for three depression classes (valance, arousal, and depression). Different colors indicate varying signal intensity levels, where darker shades represent lower values and brighter shades represent higher values.

Besides providing erroneous documents, the information has to be ready for our inference method. This section describes the methods used to generate the fundamental signal properties required for deductions, and the output depictions are shown in Figure 8. Furthermore, we justify the alteration of temporal-series signals in image time depictions and domain recurrence in the subsequent arguments [25]. Initially, the architectures of neural networks have been enhanced for computer vision and obtained excellent results in categorizing effectiveness by virtue of exploiting translational consistency via automatic feature maps, obtaining fields of reception as well as learning by weight sharing through considering signals in a manner to take advantage of the DL ability. Second, a single visual representation with specified dimensions is capable of recording both temporal and spatial properties for a period of time equivalent to a single event prior to the ESM valence and arousal assessment [26,27]. Furthermore, it includes elements of intensity and average strength, and variable ranges at multiple time points in an individual window can enhance categorization accuracy. Finally, comparable algorithms are used to capture temporal and frequency connections for recognizing human behavior through sensor data and encourages and supports the exploration.

#### 2.3.1. Multi-Resolution Short-Time Fourier Transform (MR-STFT)

MR-STFT seeks to enhance time-frequency resolution of standard STFT through continuously altering the window size based on the signal frequency content [28]. The MR-STFT mathematical representation details are provided as follows: The input size is represented as y(ti) and the time-domain window function is denoted as x(ti,fe), which is frequency reliant and the window size changes for various frequencies. The equation of MR-STFT is provided below:

(1) $$MR\text{-}STFT\left\{y\left(ti\right)\right\}\left(ti,fe\right)=\int_{-\infty }^{\infty }y\left(\tau \right)\omega \left(\tau -ti;fe\right)d^{-k2\pi g\tau }e\tau$$

#### 2.3.2. Wavelet Packet Transform (WPT)

WPT is an expansion of the Discrete Wavelet Transform (DWT), where the signal is split into both estimate and detail components at every level [29]. The signal in WPT is recursively split into sub-bands at every level and creates a full decomposition of low and high frequency components. The WPT mathematical equation is as follows:

(2) $$y\left(ti\right)=\sum_{l}b_{m,l}\psi_{m,l}\left(ti\right)$$

(3) $$Approximation\;Coefficients=\sum_{l}y\left(ti\right)\phi_{m,l}\left(ti\right)$$

(4) $$Detail\;Coefficients=\sum_{l}y\left(ti\right)\phi_{m,l}\left(ti\right)$$

#### 2.3.3. Multiscale Hilbert–Huang Transform (HHT)

This Multiscale HHT encompasses the traditional HHT through applying a large signal scale to extract relevant features within various frequency bands and timescales [30]. Moreover, traditional HHT contains two phases: first, Empirical Mode Decomposition (EMD) decomposes a signal into Intrinsic Mode Functions (IMFs). A second phase, Hilbert Spectral Analysis (HSA), applies the Hilbert transform to every IMF to attain time-frequency-energy representations. To a single y(ti), the EMD process decomposes it in a sum of IMFs b_j (ti) and re(ti) a residue:

(5) $$y\left(ti\right)=\sum_{j=1}^{M}b_{j}\left(ti\right)+re\left(ti\right)$$

(6) $$x_{j}\left(ti\right)=b_{j}\left(ti\right)+i{\hat{b}}_{j}\left(ti\right)=C_{j}\left(ti\right)g^{i\theta_{j}\left(ti\right)}$$

(7) $$\omega_{j}\left(ti\right)=\frac{e\theta_{j}\left(ti\right)}{eti}$$

(8) $$y\left(ti\right)\to \{y_{1}\left(ti\right),y_{2}\left(ti\right),\ldots ,y_{i}\left(ti\right)\}$$

(9) $$y_{i}\left(ti\right)=\sum_{j=1}^{M_{i}}b_{j,i}\left(ti\right)+re_{i}\left(ti\right)$$

(10) $$x_{j,i}\left(ti\right)=b_{j,i}\left(ti\right)+{\hat{b}}_{j,i}\left(ti\right)=C_{j,i}\left(ti\right)g^{i\theta_{j,i}\left(ti\right)}$$

(11) $$\omega_{j,i}\left(ti\right)=\frac{e\theta_{j,i}\left(ti\right)}{eti}$$

Here, the multiscale time-frequency representation incorporates the outcomes within all scales to create an extensive time-frequency depiction which gathers signal features within different resolutions.

### 2.4. Self-Supervision-Enabled Compounded Multi-Modal Feature-Learning Network

In this research, we design an S2-CFL strategy for quantifying and classifying depressive states and emotions into a binary classification problem as low/high depressive states, low/high arousal, and low/high valance. Whereas emotion states are classified into various classes such as happy, angry, sad, excited, etc. …, The designed S2-CFL model is composed of two modules with feature fusion and an attention block. The two modules are named Triple-Path Encode–Decoder Network (TP-EDN), and Densely Connected Convolution Pyramidal Transformer Network (DC2-PTN). Our work processes both the raw signal inputs (i.e., HR, GSR, and ACC), and also transforms then into image-like transformations using MR-STFT, WPT, and HHT. The raw signal input is accepted by the TP-EDN, and the signal-image transformations are accepted by the DC2-PTN. The TP-EDN is first pretrained using a reconstruction loss (Mean Squared Error) to learn latent representations, after which the encoder weights are fine-tuned for downstream classification tasks.

#### 2.4.1. Triple-Path Encoder–Decoder Network (TP-EDN)

In order to process the raw Heart Rate (HR), Galvanic Skin Response (GSR), and Accelerometer (ACC) data for depressive state quantification, this research designs a TP-EDN. As the name suggests, each of the paths will be devoted to one modality (i.e., ACC, GSR, and HR) for feature extraction in independent fashion. The design of the TP-EDN is provided as follows:

**Triple-Path Encoder Network:** The proposed research designs a separate encoder network for each modality ($y_{HR},y_{GSR},y_{ACC}$). The designed encoders are responsible for extracting the frequency and temporal features from convolution and recurrent layers respectively. Let $y_{HR}\left(t\right)$ denote the input HR signal over time *t*. The encoder for HR utilizes convolution and LSTM layers for extracting temporal and spatial features, respectively. The equation for the HR encoder is denoted as

(12) $$hid_{HR}^{\left(l\right)}=LSTM^{\left(l\right)}\left(\gamma \left(We_{HR}^{\left(l\right)}*y_{HR}+Bi_{HR}^{\left(l\right)}\right)\right)$$

In a similar manner, both the GSR and ACC data features are extracted by series of convolution and LSTM layers, respectively. The formulation of those encoders are denoted by

(13) $$hid_{GSR}^{\left(l\right)}=LSTM^{\left(l\right)}\left(\gamma \left(We_{GSR}^{\left(l\right)}*y_{GSR}+Bi_{GSR}^{\left(l\right)}\right)\right)$$

(14) $$hid_{ACC}^{\left(l\right)}=LSTM^{\left(l\right)}\left(\gamma \left(We_{ACC}^{\left(l\right)}*y_{ACC}+Bi_{ACC}^{\left(l\right)}\right)\right)$$

**Feature Fusion:** Once the three modality signals are separately encoded, the extracted features are fused using a concatenation mechanism. The feature outputs from the encoders are denoted as $hid_{HR},hid_{GSR},hid_{ACC}$. The formulation of feature fusion using the concatenation mechanism is defined by

(15) $$hid_{fu}=Den\left(\left[hid_{HR},hid_{GSR},hid_{ACC}\right]\right)$$

**Triple-Path Decoder Network:** The fused raw spatial and temporal features are interpreted and reconstructed using a decoder network for quantifying the depressive states. All the three paths of decoder output for three varied depressive tasks (i.e., depression, arousal, and valance). The mathematical formulations of feature reconstruction for depressive states are provided as follows

(16) $${\hat{x}}_{val}=Smax\left(Den_{val}\left(hid_{fu}\right)\right)$$

(17) $${\hat{x}}_{aro}=Smax\left(Den_{aro}\left(hid_{fu}\right)\right)$$

(18) $${\hat{x}}_{dep}=Smax\left(Den_{dep}\left(hid_{fu}\right)\right)$$

#### 2.4.2. Densely Connected Convolution Pyramidal Transformer Network (DC2-PTN)

**Figure 9:**
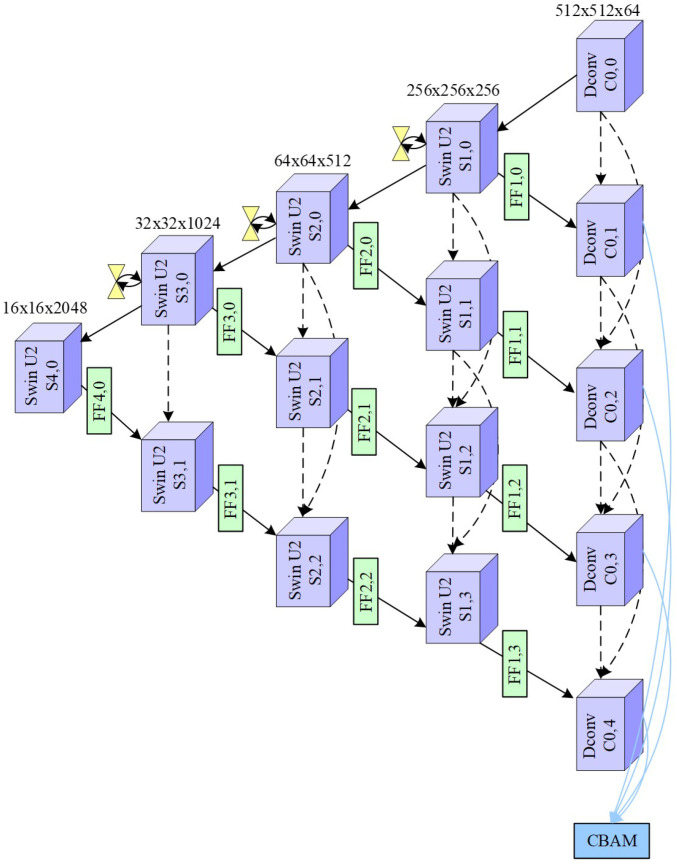
Illustration of DC2-PTN model for feature correlation.

The DC2-PTN module is designed to enhance feature representation through hierarchical and multiscale learning in which the backbone transformer extracts coarse spatial-temporal representations from signal-to-image inputs whereas the Mixed Feature Pyramid (MFP) module performs feature refining by enabling interactions across multiple scales that allow the model to capture both fine-grained and global patterns. In addition to that, the dense convolutional connections are employed for performing feature reuse and stabilizing gradient propagation, which ensures that both shallow and deep features contribute effectively to the final representation. For integration, we employ feature fusion blocks that integrate outputs from different encoder stages, which aligns complementary information from physiological and transformed inputs perfectly. At last, the CBAM is applied to emphasize informative regions and suppress irrelevant features, which leads to enhanced depressive state discrimination. The illustration of the designed DC2-PTN is shown in Figure 9. The designed model is elaborated based on the Algorithm 1. Initially, we perform parameter *j* and *i* initialization to satisfy the conditions as follows:

(19) 1≤j≤5,j∈M

(20) 0≤i≤4,i∈M

(21) i=0,j∈{1,2,3,4,5}

(22) i=1,j∈{1,2,3,4}

(23) i=2,j∈{1,2,3}

(24) i=3,j∈{1,2}

(25) i=4,j∈{1}

After that, we obtain encoder features for compensating for the details missing from the main network as

(26) $$U_{1}^{\left(en_{1,2}\right)},U_{2}^{\left(en_{2,3}\right)}=TPEDN\text{\_}SSL\left({\hat{x}}_{val},{\hat{x}}_{aro},{\hat{x}}_{dep}\right)$$

In the parallel path, the first column features are generated as

(27) $$DC_{0,0},Sw_{j,i=0}^{\prime }=SwU2\text{\_}backbone\left(DConv\left(CC\left({\hat{x}}_{val},{\hat{x}}_{aro},{\hat{x}}_{dep}\right)\right)\right)$$

The attained and correlated features are estimated using a Mixed Feature Pyramid (MFP) network, which ensures intra- and inter-scale interaction information as

(28) $$Sw_{1,0},Sw_{2,0},Sw_{3,0}=MFP\left(Sw_{1,0}^{\prime },Sw_{2,0}^{\prime },Sw_{3,0}^{\prime }\right);\;Sw_{4,0}=Sw_{4,0}^{\prime }$$

The remaining transformers and fused features in the 1 to 5 rows can be generated as

(29) $$Sw_{j,1}=SwU2\left(Con\left(CC\left(Sw_{j,0},Vq\left(FF_{j+1,0}\right)\right)\right)\right)$$

(30) $$Sw_{j,2}=SwU2\left(Con\left(CC\left(Sw_{j,0},Sw_{j,1},Vq\left(FF_{j+1,1}\right)\right)\right)\right)$$

(31) $$Sw_{j,3}=SwU2\left(Con\left(CC\left(Sw_{j,0},Sw_{j,1},Sw_{j,2},Vq\left(FF_{j+1,2}\right)\right)\right)\right)$$

(32) $$FF_{j,i}=FF\left(Sw_{j,i},U_{1}^{\left(en_{1,2}\right)},U_{2}^{\left(en_{2,3}\right)}\right)$$

The remaining DCon features are acquired as

(33) $$DC_{0,1}=DCon\left(CC\left(DC_{0,0},FF_{1,0}\right)\right)$$

(34) $$DC_{0,2}=DCon\left(CC\left(DC_{0,0},DC_{0,1},FF_{1,1}\right)\right)$$

(35) $$DC_{0,3}=DCon\left(CC\left(DC_{0,0},DC_{0,1},DC_{0,2},FF_{1,2}\right)\right)$$

(36) $$DC_{0,4}=DCon\left(CC\left(DC_{0,0},DC_{0,1},DC_{0,2},DC_{0,3},FF_{1,3}\right)\right)$$

(37) $$DC_{0,5}=DCon\left(CC\left(DC_{0,0},DC_{0,1},DC_{0,2},DC_{0,3},DC_{0,4},FF_{1,4}\right)\right)$$

**Algorithm 1** Depressive State Quantification (DSQ) using DC2-PTN1:**Input:** image-signal feature pairs2:**Output:** DSQ3:Initialize j,i and satisfy Equations (19)–(25)4:Attain features from TP-EDN and perform update $U_{1}^{\left(en_{1,2}\right)},U_{2}^{\left(en_{2,3}\right)}$ using ${\hat{x}}_{val},{\hat{x}}_{aro},{\hat{x}}_{dep}$ from Equation (26)5:Attain features from main column and perform update $DC_{0,0},Sw_{\left(j,i=0\right)}^{\prime }$ using ${\hat{x}}_{val},{\hat{x}}_{aro},{\hat{x}}_{dep}$ from Equation (27)6:Utilize MFP for feature estimation and perform update $Sw_{1,0},Sw_{2,0},Sw_{3,0},Sw_{4,0}$ by simplifying Equation (28)7:Attain features from rows 1 to 58:**for** i=0 to 4 **do**9:      **if** i==1 **then**10:          Calculate $Sw_{\left(j,1\right)}$ by $Sw_{\left(j,0\right)},FF_{\left(j+1,0\right)}$ based on Equation (29)11:    **else if** i==2 **then**12:          Calculate $Sw_{\left(j,2\right)}$ by $Sw_{\left(j,0\right)},Sw_{\left(j,1\right)},FF_{\left(j+1,1\right)}$ based on Equation (30)13:    **else if** i==3 **then**14:          Calculate $Sw_{\left(j,3\right)}$ by $Sw_{\left(j,0\right)},Sw_{\left(j,1\right)},Sw_{\left(j,2\right)},FF_{\left(j+1,2\right)}$ based on Equation (31)15:    **else if** i==4 **then**16:          Calculate $Sw_{\left(j,4\right)}$ by $Sw_{\left(j,0\right)},Sw_{\left(j,1\right)},Sw_{\left(j,2\right)},Sw_{\left(j,3\right)},FF_{\left(j+1,3\right)}$ based on Equation (32)17:    **end if**18:    Perform Update of $FF_{\left(j,i\right)}$ from Equations (33)–(37)19:**end for**20:Calculate $DC_{0,1},DC_{0,2},DC_{0,3},DC_{0,4},DC_{0,5}$ by estimating DCon features from Equations (33)–(37)21:Quantify DSQ by updating DSQ from Equation (38)

At last, the depressive state quantification (DSQ) is achieved by fusing the encoder feature pair and image pair features as

(38) $$DSQ=Sig(Con_{1\times 1}\left(CBAM\left(CC\left(DC_{0,1},DC_{0,2},DC_{0,3},DC_{0,4},DC_{0,5}⊕\left({\hat{x}}_{val},{\hat{x}}_{aro},{\hat{x}}_{dep}\right)\right)\right)\right))$$

Overall, the proposed S2-CFL shows better performance by incorporating parallel encoder–decoder architectures. At first, the TP-EDN is composed of parallel encoder–decoder networks for extracting the multiscale raw features. Secondly, the DC2-PTN is responsible for extracting the spatial and temporal features by ensuring the inter- and intra-scale interactions. Those combinations help the designed model to perform accurate depressive state quantifications.

### 2.5. Fine-Grained Emotion Classification Network

In this research, we utilize three standard Machine Learning (ML) algorithms, CatBoost (CB), eXtreme Gradient Boosting (XGB), and Random Forest (RF), with an explainability method, as a fine-grained emotion classification network. These algorithms are completely self supervised and reduce complexity and bias variance, even though the data is voluminous and have unique samples. Furthermore, the explainability concept resolves the ‘black box’ nature of conventional ML algorithms by providing reliable emotion classification results in multiple classes such as happy, sad, angry, calm, tired, etc. With the five-fold classification approach, we analyze the performance of the model and divide the training and test sets in a ratio of 80% and 20%, respectively, using a stratified sampling method. The detailed mathematical modeling of FGECN is provided as below:

Initially, the input feature vectors, target labels, and prediction condition are assigned. The input feature vector is represented by

$$Y=\{y_{1},y_{2},\ldots ,y_{m}\}$$

in which *Y* denotes the wearable sensor input and psychological self-report features. The target labels are denoted by

$$X=\{x_{1},x_{2},\ldots ,x_{n}\}$$

in which *X* denotes the categories of emotions such as angry, sad, happy, calm, excited, etc. …, The model prediction can be formulated as

$$\hat{X}=f\left(Y\right),$$

where *f* denotes the ML model utilized for fine-grained emotion classification. In addition to that, we also introduce the explainability mapping function Ex for correlating the *Y* to the $\hat{X}$.

For every prediction ${\hat{x}}_{j}$, the ML model allocates an importance score to the feature vectors. The feature contribution of $y_{i}$ to the ${\hat{x}}_{j}$ can be formulated as

(39) $$A_{ji}=Ex\left(f\left(Y\right),y_{i}\right)$$

From the above equation, the feature $y_{i}$ importance score is denoted by $A_{ji}$ for the prediction ${\hat{x}}_{j}$. The model predictions of CatBoost (CB), eXtreme Gradient Boosting (XGB), and Random Forest (RF) are denoted as

(40) $${\hat{X}}_{CB}=w_{CB}f_{CB}\left(Y\right)+w_{XGB}f_{XGB}\left(Y\right)+w_{RF}f_{RF}\left(Y\right)$$

From the above equation, $w_{CB}$, $w_{XGB}$, and $w_{RF}$ denote the weights of the ML model that are determined by the regularized feature contribution scores as

(41) $$\begin{array}{cc} w_{CB} & =\frac{\sum_{i=1}^{m}A_{ji}^{CB}}{\sum_{k\in \{CB,XGB,RF\}}\sum_{i=1}^{m}A_{ji}^{k}},\;w_{XGB}=\frac{\sum_{i=1}^{m}A_{ji}^{XGB}}{\sum_{k\in \{CB,XGB,RF\}}\sum_{i=1}^{m}A_{ji}^{k}},\\ w_{RF} & =\frac{\sum_{i=1}^{m}A_{ji}^{RF}}{\sum_{k\in \{CB,XGB,RF\}}\sum_{i=1}^{m}A_{ji}^{k}} \end{array}$$

where the explainability of every feature $y_{i}$ is represented by the contribution of scores $A_{ji}^{k}$ to model *k*. This research also adopts a loss function into the explainable ML to penalize the prediction which can diverge from the interpretable results. The loss function can be formulated as

(42) $$Lo=Lo_{cla}+N\sum_{j=1}^{n}\left(\sum_{i=1}^{m}\left|A_{ji}-Ex\left(A_{ji}\right)\right|\right)$$

where $Lo_{cla}$ denotes the cross-entropy classification loss function, the explainability impact can be regularized by the hyperparameter N, and the expected importance score for the $y_{i}$ is denoted by $Ex(A_{ji})$.

#### Implementation and Reproducibility Analysis

To ensure the reproducibility of the proposed S2-CFL framework, this study provides detailed information regarding model training and implementation in which the model was implemented from a deep learning framework with standard optimization techniques. The training process utilized the Adam optimizer with a learning rate of 0.001 and a batch size of 32, and training was conducted for 50 epochs. In order to prevent overfitting, we employed early stopping in which all input signals were normalized using Z-score normalization, and stratified 5-fold cross-validation was adopted to ensure robust evaluation. By employing a grid search, the ML model’s hyperparameters were tuned. The complete implementation details, including model architecture configurations and preprocessing steps, will be made publicly available to support reproducibility and further research.

## 3. Results

In this section, we explain the experimental results for depressive state severity quantification for every depression category, and emotion classification with unimodal and multimodal categories. With the performance metrics accuracy, specificity, sensitivity, and F1-score, the proposed performance was analyzed.

### 3.1. Comparison with the Existing Works

To validate the effectiveness of the proposed S2-CFL framework, this study compares the proposed performance with several baseline and existing approaches that are commonly utilized for depression and emotion classification using wearable and physiological data. The selected baselines include traditional machine learning models such as Random Forest (RF) and eXtreme Gradient Boosting (XGBoost) and deep learning models including Convolutional Neural Networks (CNNs) and Long Short-Term Memory (LSTM) networks. More clearly, the traditional ML models are trained on handcrafted statistical features extracted from physiological signals, whereas the deep learning models operate on either raw signals or signal-to-image representations. In contrast, the proposed S2-CFL integrates both raw and transformed representations through a self-supervised multimodal learning strategy.

#### Evaluation Metrics

In order to ensure the performance evaluation correction, this research employs the metrics below:

(i) Accuracy: The overall classification correctness is measured by accuracy, which can be formulated as
  (43) Acc=(TP+TN)/(TP+TN+FP+FN)
(ii) Sensitivity: This is also known as recall, which computes the ability to correctly identify the positive instance and can be formulated as
  (44) Sensitivity=TP/(TP+FN)
(iii) Specificity: This measures the ability to correctly identify the negative instance and can be formulated as
  (45) Specificity=TN/(TN+FP)
(iv) F1-Score: The F1-score shows the harmonic mean of the recall and precision and can be formulated as
  (46) F1-Score=2×(Precision×Recall)/(Precision+Recall)

### 3.2. Depressive State Severity Quantification Analysis

In order to examine the efficiency of the proposed S2-CFL model, we examine the three signal-to-image transforms MR-STFT, WPT, and HHT with every modality (HR, GSR, and ACC). The average accuracy and F1-score achieved are 62.99±0.68 and 62.89±0.41, which are shown in Table 2. The results shows that the model shows balanced performance with lesser overfitting, with average numerical results in terms of accuracy, sensitivity, specificity, and F1-score values of 61.44±2.26, 67.83±2.87, 54.24±2.89, and 61.13±2.22, respectively.

Furthermore, in Table 3 and Table 4, it is clearly indicated that the arousal and valance states tend to highly overfit in unimodal cases (i.e., fluctuating constantly), with average performance of sensitivity and specificity of 61.71±41.58 and 51.51±41.32 respectively. In similar manner, for valance, the sensitivity and specificity achieved are 28.43±34.11, and 91.4±36.43 respectively.

The multi modality effect in the three depressive states depression, valance, and arousal are quantified in Table 5, Table 6 and Table 7. The performance for the depression state in terms of average accuracy, sensitivity, specificity, and F1-score values are 58.62±2.57, 76.1±11.1, 46.1±21.1, and 61.1±1.1 respectively. On the other hand, the valance state shows average accuracy of 66.87±4.96, sensitivity of 41.86±31.78, specificity of 62.28±2.42, and F1-score of 82.68±28.89. Finally, the arousal state shows better average performance with respect to input modalities, with accuracy of 85.69±6.96, sensitivity of 96.32±8.19, specificity of 26.17±22.18, and F1-score of 62.24±2.46.

We perform additional experiments to support the depressive state quantification using the designed DL model for reducing the unwanted overfitting issues. More clearly, we employ further additions such as dense transformer, fusion modules, and attention layer. This research shows balanced performance and reduced overfitting. In addition to that, we notice that the negligible difference among those variants’ performance, especially in the 25 min time windows that contained ESM data that were not robust for quantification tasks.

On the whole, we have concluded that the multimodal inputs perform better than the unimodal inputs in all depressive cases. Whereas in the case of multimodal input, the valance data shows more reliable results than the other two cases arousal and depressive data. However, the valance data are also prone to overfitting with sensitivity, and specificity tends to 0 and 100, which indicates that the model was highly prone to false positives during the testing stage.

### 3.3. Emotion Classification Analysis

We utilize three ML algorithms, CatBoost (CB), eXtreme Gradient Boosting (XGB) machine, and Random Forest (RF), amalgamated with an explainability method for emotion classification in fine-grained fashion. All of those algorithms were selected based on its imbalanced sample handling, variance-bias trade off, complexity and ability trade off, handling of high dimensional inputs, and robustness.

With the multiple emotion classes happy, sad, relaxed, angry, calm, tired, etc., the performance of emotion classification is analyzed. Table 8 shows the quantitative analysis of the fine-grained emotion classification network under varied ML algorithm settings. From the analysis, it is inferred that the CB gains better accuracy of 93.5%, which outperforms others, especially in complex emotion classification (i.e., anger and calmness), as both are very difficult to differentiate. On the other hand, the XGB achieves accuracy of 91.9%, which shows excellent performance in capturing emotions such as sadness and happiness, in which very subtle variations are needed to be extracted. Finally, the RF shows lesser performance, with better reliability in classification of 89.9%. However, the RF effectively handled imbalanced data, especially with tired emotion classes.

**Figure 10:**
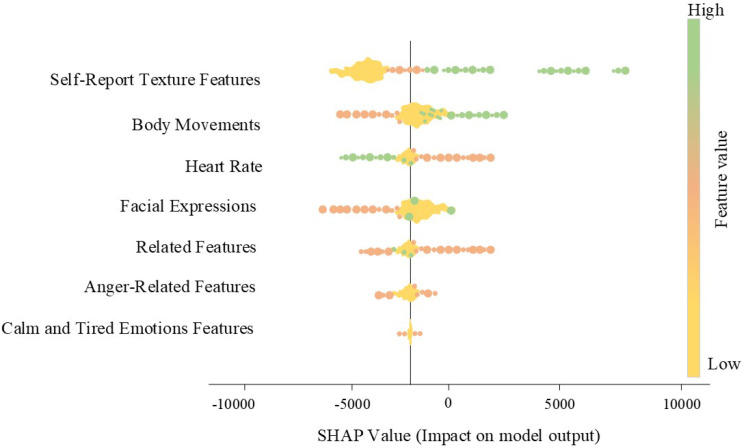
Feature contribution of ML models using SHAP.

We utilize SHapley Additive exPlanations (SHAP) for examining the explainability of the ML models, which provides feature contribution transparency for emotion categorization, as shown in Figure 10. The explainability methods are highly utilized for resolving the black box nature of the conventional ML models and also ensuring improved interpretability. In terms of feature importance, the adopted model recognizes features such as self-report texture features, sensory data (i.e., body movements and heart rate), and facial expressions, for categorizing fine-grained emotions. For instance, happiness- and anger-related features are firmly highlighted by the CB model, whereas the XGBoost model shows strengthened dependence on sensory data, especially in recognizing calm and tired emotions. Furthermore, the RF shows well-adjusted contributions among multiple features and also it is sensitive to data variance. On the whole, by analyzing the explainability methods we conclude that some of the emotion classes (i.e., tired and sad) posses overlapping natures. Especially in RF model settings, the feature importance emotion values are aligned closely, thereby showing poorer performance in explainability than the other two models.

The bias-variance trade-off among the adopted three algorithms is discussed based on the varied approaches of the ML models. The CB utilizes a gradient boosting method to ensure steady performance on both the small and larger emotion classes. In addition to that, the self-supervised nature of the CB allows it to withstand noisy labels and resolve overfitting issues. The fine-grained variations among the emotions with subtle emotional cues are excellently captured by the XGBoost model, which is also prone to the variance problem. Finally, the RF model is highly prone to the bias problem as it provides decisions by amalgamating from the multiple decision tress and also handling larger datasets. The variance is lesser but the chance of overfitting is higher, particularly for classes like happiness, excitement, etc.

In summary, the CB performed well for happy and angry emotions where the expression nuances with context information are critical, but lacked for robustness. The tiredness and calm emotions are effectively captured by the XGBoost model, which characterized physiological indicators (i.e., stillness and lower heart rate), which make it highly sensitive to sensory data. Finally, the RF model shows better performance in the sad class by handling the imbalanced data even though the classes are lesser in number.

#### Interpretability Analysis of Multimodal Features

To provide a more rigorous understanding of the model’s decision-making process, we analyze feature contributions using SHAP-based explainability across different data modalities, including physiological signals (HR, GSR, and ACC) and psychological self-reports. From Figure 10, it can be observed that HR features show strong positive contributions toward high-arousal emotional states, indicating their relevance in capturing physiological activation. The GSR contributes significantly to stress-related emotions such as anxiety and tension, reflecting autonomic nervous system responses. The ACC features are more influential in distinguishing low-valence states as shown in Table 9 (e.g., fatigue and inactivity), capturing behavioral and movement-related patterns. Furthermore, the model demonstrates consistent feature attribution patterns across different emotion classes: emotions such as anger and excitement are primarily influenced by high physiological activation (HR and GSR). Calm and tired states show stronger dependence on ACC and lower HR variability. Sadness-related states exhibit overlapping contributions from both physiological and self-reported features, explaining the moderate classification difficulty observed. These findings confirm that the proposed multimodal framework effectively integrates heterogeneous data sources and that the model’s predictions are aligned with known physiological and psychological patterns. This strengthens the interpretability and reliability of the proposed approach.

### 3.4. Ablation Study

An ablation analysis is conducted to examine the individual contribution of every component by systematically enabling and disabling key modules of the architecture. By this analysis, we focus on understanding the raw signal learning, image-based representation, feature fusion, and attention mechanisms that influence overall performance.

From the ablation analysis results as shown in Table 10, it is confirmed that the TP-EDN module alone provides stable performance by capturing temporal dependencies from raw physiological signals. Furthermore, the DC2-PTN module improves spatial-temporal representation through signal-to-image transformations, which provides better performance than TP-EDN. When integrating both the TP-EDN and DC2-PTN, there is significant enhancement in performance which complements both the transformed and raw representations. The information loss reduction and multimodal feature alignment are achieved by employing a feature-fusion technique. Finally, the utilization of CBAM ensures attention capability by focusing only on discriminative features, which results in better overall performance.

### 3.5. Statistical Validation

In order to examine the robustness and reliability of the proposed S2-CFL framework, we performed statistical validation using stratified five-fold cross-validation, which ensures that each fold preserves the class distribution and reduces bias due to data imbalance. The performance metrics, including accuracy and F1-score, are reported as mean ± standard deviation across the folds. Table 2, Table 3 and Table 4 show that the proposed model achieves consistent performance and with relatively low variance, with accuracy of 61.44 ± 2.26 and an F1-score of 61.13 ± 2.22, indicating stable learning behavior across different data splits. To further assess the significance of performance improvements, a paired statistical comparison between the proposed S2-CFL model and baseline methods was performed as shown in Table 11. A paired t-test was employed on the fold-wise results and the obtained p-values were below 0.05, which confirms that the improvements are statistically significant. In addition to that, the consistency of the model predictions was also analyzed by examining the variation in sensitivity and specificity for the minor fluctuations observed due to class imbalance and temporal variability in ESM data. However, the overall trend remained stable, which supports the generalization capability of the model. From the results, it can be seen that the proposed S2-CFL framework provides reliable and reproducible performance. Also, note that the observed improvements are not due to random variation but are statistically meaningful.

## 4. Discussion

The purpose of this study is to differentiate the behaviors of depressed individuals along with emotional stimuli and possibly to realize emotional triggers in response to authentic procedures. Each person plotted in this dataset showed high-intensity emotional states and incidence of depression, as formerly specified. This demands future research into how depression-related strong or weak emotions respond to stimuli or experiences. Depression causes obvious defective regulation of affective experience and affective quality assessment, according to [31]. The neutral arousal/valence state is typically the most commonly experienced [32] because of poor emotional modulation to affective cues.

The attained performance metrics were dependable and achieved the detection of depression severity by employing wearable devices with participants with similar demographic features such as age, race and educational attainment [33]. It appeared that maximum levels of depression symptoms were associated with higher variations in heart rate at night between 4 am and 6 am in morning. There were also strong associations between harshness and weekday daily activity patterns. Therefore, the broad continuous diurnal and nocturnal biomarkers measured in [34] were supported by our findings from studying individuals through conscious ESM activities.

Higher levels of depression were demonstrated in low-arousal states that were connected to low valence fine-grained emotions such as exhaustion, melancholy or lethargy. This is because of the circumstances in which high arousal occurs while the cortical circuits of brain are active in response to certain stimuli [35]. However, the reverse of these results supported the theory that either self-reported BDI-II scores were not revealing of honest primary mental states or that self-reported valence and arousal were deliberately misplaced to be more positive. A reasonable psychological connection which can reconcile our findings with previous research is the impression of ambivalence regarding emotional communication. People with this disorder will be disposed to avoiding articulating their emotions [36] due to the effects of depression. It was shown in [37] that memory tests for individuals with heightened sadness can encourage high-arousal states and increase their intention to pursue support. It was probable that during learning, the stimuli or experiences which contributed to the creation of the DAPPER dataset were encountered in surroundings which were comforting or supportive.

**Figure 11:**
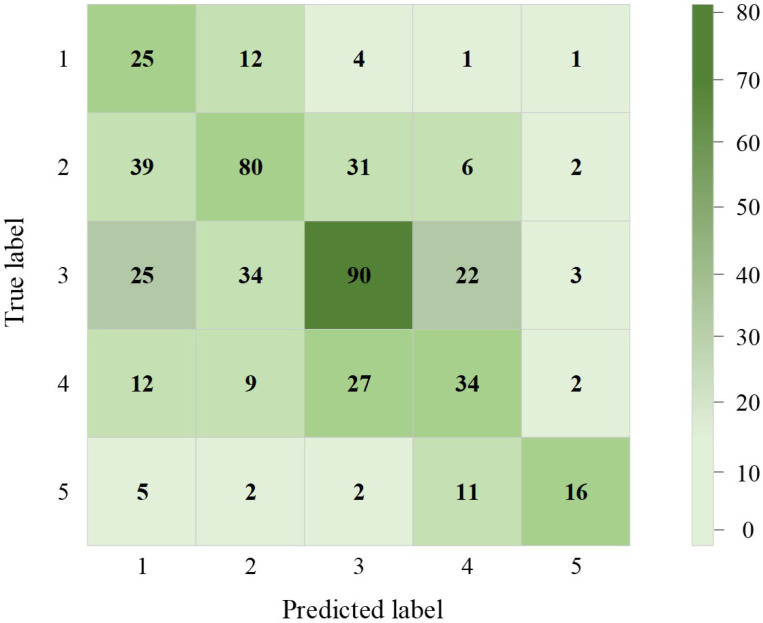
Confusion matrix representation of five emotional classes.

When there were erroneous values, noise saturation and uncalibrated errors, it was accompanied by typical patterns connected to detailed physical/mental illnesses, which will actually be imitated because of the diverse nature of numerous consumer-grade wearables [38]. Moreover, it was not identified whether the participants were receiving prescribed antidepressants, psychiatric counselling or any kind of treatment that would cause confusing variables which cannot be measured. Figure 11 shows the confusion matrix representation of emotional classes.

Its employment in biomedical domains has been delayed due to restraints and problems caused by class imbalances and the difficulty of data collection, while DL has displayed excellent outcomes in a variety of sectors and topical research. DL approaches normally recognize inaccurate relationships in training data for clinical inquiries by exploiting biological cues, according to arguments made in [39]. Our conclusions are made in the context of our study because some emotional states are implicit, such as depression, valence or arousal, and do not illustrate all participants in the same way or to the same extent [40]. To overcome the relative sparsity of wearable measurements that remains, future research could examine the ratio of the duration of normal physiological behavior and the duration of context-related instantaneous responses to exact stimuli and sophisticated performance scores. It would appear that larger volumes of data from wearables are essential.

According to [41], the approaches show the superiority of popular groups due to their higher prior possibility of dealing with confused information. In contrast to summary measures like wavelet decomposed features, more sophisticated strategies are required to account for a thoughtful grasp of morphology and patterns, while enhancing continuous raw signals and rectifying class imbalances [42]. We contend that, as established in [43], the lightweight approaches DWT + conventional ML were more suitable for power-efficient placement on wearables or edge devices [44,45].

We propose utilizing this research to assess the findings regarding depression and transient emotional states, as well as to find a baseline for performance benchmarking on the DAPPER dataset.

## 5. Conclusions

This study presents a Self-Supervision-Enabled Compounded Multi-Modal Feature-Learning Network (S2-CFL) for depressive state detection and fine-grained emotion classification using wearable sensor data and psychological self-reports. The proposed framework effectively combines temporal and spatial feature learning through TP-EDN and DC2-PTN modules, followed by multimodal fusion and supervised emotion classification. Experimental results demonstrate that multimodal learning improves performance compared to unimodal approaches, while also providing interpretable insights into emotional and physiological patterns. However, challenges such as class imbalance, limited dataset size, and overfitting in certain scenarios highlight the need for further improvements. Future research should focus on enhancing generalization, incorporating larger and more diverse datasets, and developing efficient models for real-world mental health monitoring applications.

## Figures and Tables

**Table 1:** Details of DAPPER Dataset.

Data	Description
**Psychological Data (DRM and ESM)**	
DRM	The DRM data includes day-to-day activities and how the patients experience emotions over the time.
ESM	This includes emotionally supported actions and sentiments over the time.
**Physiological Data (ACC Data, GSR, and HR)**	
ACC	The ACC data is acquired using a triaxial accelerometer that shows changes in speed in relation to cartesian coordinates.
GSR	The GSR data is obtained through electrodermal activity.
PPG	The PPG data is obtained by photoplethysmography signals.

**Table 2:** Roles and contributions of model components.

Component	Role	Contribution
Swin Transformer Backbone	Extracts hierarchical temporal features	Captures long-range dependencies
Mixed-Feature Pyramid	Multiscale feature interaction	Enhances robustness across scales
Dense Convolution	Feature reuse and propagation	Reduces vanishing gradient problem
Feature Fusion	Integrates multimodal features	Improves complementary information
CBAM Attention	Focus on important regions	Enhances classification performance

**Table 3:** Performance comparison of different methods.

Method	Accuracy (%)	F1-Score (%)
Random Forest (RF)	89.9	90.4
XGBoost (XGB)	91.9	91.4
CNN	88.4	87.9
LSTM	86.5	85.8
Multimodal DL	89.2	88.6
Proposed S2-CFL	93.5	92.8

**Table 4:** Performance of model on depression state with image transforms.

Method	Accuracy	Sensitivity	Specificity	F1-Score
HR				
MR-STFT	61.948	67.449	55.766	61.508
WPT	62.878	61.370	55.395	62.883
HHT	58.860	65.643	55.899	58.870
GSR				
MR-STFT	62.388	63.858	58.948	62.483
WPT	61.845	65.564	58.247	61.886
HHT	51.324	65.198	54.817	59.887
ACC				
MR-STFT	61.257	64.738	57.372	58.855
WPT	58.866	67.120	53.956	58.538
HHT	63.518	69.856	56.683	63.279

**Table 5:** Performance of model on arousal state with image transforms.

Method	Accuracy	Sensitivity	Specificity	F1-Score
HR				
MR-STFT	67.159	93.219	25.553	59.367
WPT	69.618	97.751	25.272	61.392
HHT	68.829	97.615	22.869	58.242
GSR				
MR-STFT	68.142	92.491	29.115	58.783
WPT	71.139	91.415	24.276	62.345
HHT	68.649	99.858	23.833	61.846
ACC				
MR-STFT	76.255	19.287	84.492	61.898
WPT	75.988	16.211	85.598	58.886
HHT	76.448	17.182	85.769	61.485

**Table 6:** Performance of model on valance state with image transforms.

Method	Accuracy	Sensitivity	Specificity	F1-Score
HR				
MR-STFT	92.846	89.117	14.836	61.977
WPT	92.526	88.814	11.621	61.218
HHT	92.378	88.689	11.385	58.847
GSR				
MR-STFT	91.397	88.355	11.968	58.663
WPT	92.281	88.318	12.554	61.437
HHT	92.626	88.929	11.695	61.312
ACC				
MR-STFT	61.582	58.539	62.814	61.677
WPT	58.998	57.324	58.955	59.138
HHT	57.818	56.728	58.868	57.899

**Table 7:** Multi-modal comparison of proposed work on depression state.

Model	Method	Acc.	Sen.	Spec.	F1
**HR & ACC**					
TP-EDN	MR-STFT-WPT	61.548	91.111	31.111	61.111
**HR & GSR**					
TP-EDN	MR-STFT-HHT	59.687	71.111	51.111	61.111
**GSR & ACC**					
TP-EDN	WPT-HHT	58.895	71.111	51.111	61.111
**HR & GSR & ACC**					
TP-EDN	MR-STFT-WPT-HHT	62.147	71.111	51.111	61.111
TP-EDN+DT	MR-STFT-WPT-HHT	61.387	59.458	63.591	61.424
TP-EDN+DT+Fusion	MR-STFT-WPT-HHT	58.278	59.343	61.488	58.427
TP-EDN+DT+Fusion+Att	MR-STFT-WPT-HHT	59.834	58.172	59.439	59.785

**Table 8:** Multi-modal comparison of proposed work on arousal state.

Model	Method	Acc.	Sen.	Spec.	F1
**HR & ACC**					
TP-EDN	MR-STFT-WPT	64.356	74.589	48.185	61.397
**HR & GSR**					
TP-EDN	MR-STFT-HHT	64.698	71.654	53.619	62.637
**GSR & ACC**					
TP-EDN	WPT-HHT	66.174	73.449	54.521	63.985
**HR & GSR & ACC**					
TP-EDN	MR-STFT-WPT-HHT	72.664	211.111	11.111	61.111
TP-EDN+DT	MR-STFT-WPT-HHT	65.877	83.383	38.915	61.159
TP-EDN+DT+Fusion	MR-STFT-WPT-HHT	65.292	84.373	34.823	59.698
TP-EDN+DT+Fusion+Att	MR-STFT-WPT-HHT	66.373	84.589	37.371	58.989

**Table 9:** Multimodal comparison of proposed work on valence state.

Model	Method	Acc.	Sen.	Spec.	F1
**HR & ACC**					
TP-EDN	MR-STFT-WPT	85.923	96.471	25.268	61.868
**HR & GSR**					
TP-EDN	MR-STFT-HHT	81.815	91.595	36.822	64.188
**GSR & ACC**					
TP-EDN	WPT-HHT	78.974	91.888	31.476	61.792
**HR & GSR & ACC**					
TP-EDN	MR-STFT-WPT-HHT	93.858	211.11	11.111	61.111
TP-EDN+DT	MR-STFT-WPT-HHT	92.214	88.658	3.193	58.925
TP-EDN+DT+Fusion	MR-STFT-WPT-HHT	91.844	87.484	6.722	61.883
TP-EDN+DT+Fusion+Att	MR-STFT-WPT-HHT	92.467	89.166	2.626	58.896

**Table 10:** Quantitative analysis of FGECN.

ML Model	Acc. (%)	Spec. (%)	Sen. (%)	F1 (%)
CatBoost	93.5	92.8	93.2	92.8
XGBoost	91.9	91.3	92.8	91.4
Random Forest	89.9	89.1	89.4	90.4

**Table 11:** Performance of different model variants.

Model Variant	Description	Accuracy (%)	F1-Score (%)
TP-EDN Only	Raw physiological signal learning	61.5	61.1
DC2-PTN Only	Signal-to-image representation learning	62.1	61.3
TP-EDN + DC2-PTN	Without fusion	65.8	62.0
+Fusion	With multimodal feature fusion	66.3	63.9
+Attention (Full S2-CFL)	Fusion + Attention (CBAM)	66.8+	70.9+

## Data Availability

The data used in this study are publicly available from previously published sources. The **Daily Ambulatory Psychological and Physiological Dataset** is available at: https://doi.org/10.6084/m9.figshare.13803185, and the **Multimodal Mixed Emotion Recognition Dataset** is available at: https://doi.org/10.5281/zenodo.11194571. Additional datasets related to emotion recognition using ECG and PPG signals are described in the literature (Refs. [21,22,23,24]).
